# Knowledge, awareness and practices of Pakistani professionals amid-COVID-19 outbreak

**DOI:** 10.1038/s41598-021-96705-w

**Published:** 2021-09-02

**Authors:** Samea Khan, Usman Shah Gilani, Syed Muhammad Muslim Raza, Tanveer Hussain

**Affiliations:** 1 Department of Molecular Biology, Virtual University of Pakistan, Lahore, Pakistan; 2 Department of Quantitative Methods, School of Business and Economics, University of Management and Technology, Lahore, Pakistan

**Keywords:** Microbiology, Molecular biology, Health care

## Abstract

End of the year 2019 marks an unprecedented outbreak of a pandemic named COVID-19 caused by the SARS-CoV-2. It was first discovered in China and later spread to the whole world, currently inflicting almost 200 countries. After China, few other countries have emerged as potential epicenters of this disease including the US, Italy, Spain and Pakistan, as indicated by the World Health Organization (WHO). Since proper preventive and curative measures in the form of a vaccine or medication are currently unavailable throughout the world, the only remedy devised to stop the spread of this virus is self-isolation. Such a measure necessitates ample awareness and understanding among people to avoid actions that lead to the spread of this virus. Pakistan is the fifth-most populous country in the world (212.2 million) and has a record of contagious outbreaks in the past. Therefore, it is key to evaluate the general understanding regarding the cause, spread and control of this disease in Pakistani population and acquire data to anticipate the possible spread and persistence of this disease to design relevant preventive measures. We have attempted to collect such data from professionals who are susceptible to acquiring the infection due to an unavoidable exposure. Keeping in view the current lock down, we have relied on an internet based collection of data by filling a self-designed questionnaire that is responded to by 1132. Descriptive and Frequency Analysis were performed on the responses received using MS Excel and SPSS software. A total of 1132 individuals responded to the questionnaire among which include academic (45.8%), non-academic (20.8%), healthcare (7.8%), security (5.9%) and other (19.7%) professionals. The questionnaire addressed the level of basic information regarding the cause, spread, cure and prevention of this disease among professionals, in an attempt to provide directions for awareness campaigns at different levels in Pakistan and provide a model for similar outbreaks in the future.To our expectations, almost after a month of the coronavirus outbreak in Pakistan, above 50% to up to 90% of the recorded responses against every question showed ample understanding regarding the cause, spread and control of the disease which is an indicator of effective public awareness campaigns throughout the country largely based on media drive.

## Introduction

Now declared a pandemic, initially COVID-19 was poorly identified as a disease caused by a novel virus and only reported as a cluster of pneumonia cases of unknown etiology from Wuhan, Hubei, Mainland China on 8th December 2019^1^. After preliminary investigation, it was believed to be caused due to a novel virus named SARS-CoV-2 that may have originated from an animal source in individuals who visited and/or consumed local seafood and animals in Wuhan^2,3^. Further spread of the disease to other countries and continents is attributed to vast traveling of non-symptomatic infected individuals, as reported by different countries^4–6^.

Currently around 200 countries and over half a million people are infected by the virus around the world. Despite rigorous efforts being put to develop a vaccine or drugs to counter coronavirus spread, current coping strategies are limited to self-isolation and general lock-downs. In this regard, Pakistan is facing a unique challenge due to poor screening capacity and consequential delay in implementing preventive measures, therefore apprehension that Pakistan is emerging as the next epicenter of this pandemic is expressed^7,8^. Awareness level and compliance of professionals play an important role in the effective and timely prevention and control of a public health crisis especially considering their increased susceptibility due to higher level of exposure to the environment and person-to-person interaction^9^. As a result, the National Action Plan for COVID-19 has been proposed by the Ministry of Health Services, Regulation and Coordination, Government of Pakistan for infection control, and a media campaign for generating awareness among the public has been initiated^10^. To bring about a comprehensive policy, teams from multi-disciplinary backgrounds are engaged including key frontlines to ensure effective their functioning^11^.

Keeping in view the importance of awareness and primary data that could help devise further control strategies, in this study, we have mainly aimed at evaluating the current level of awareness among professionals in Pakistan with regard to the cause, spread and cure of COVID-19. We have applied Descriptive and Frequency Analysis on data collected through an online medium by a self-designed questionnaire.

## Results

### Demographic distribution

**Figure 1:**
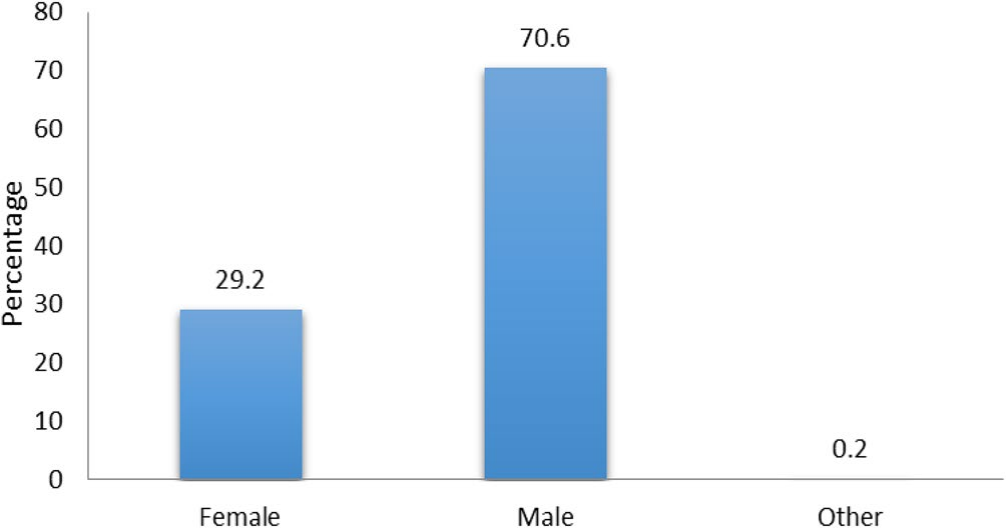
Percentage of gender wise responses. The bar chart represents percentages of respondents belonging to different genders.

**Figure 2:**
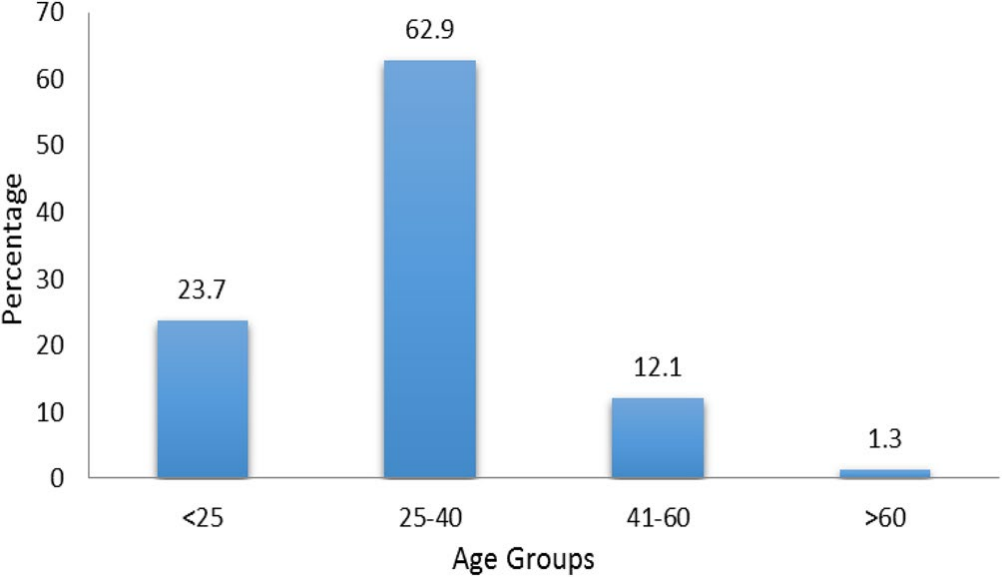
Percentage of age wise responses. The bar chart represents percentages of respondents belonging to different age groups.

**Figure 3:**
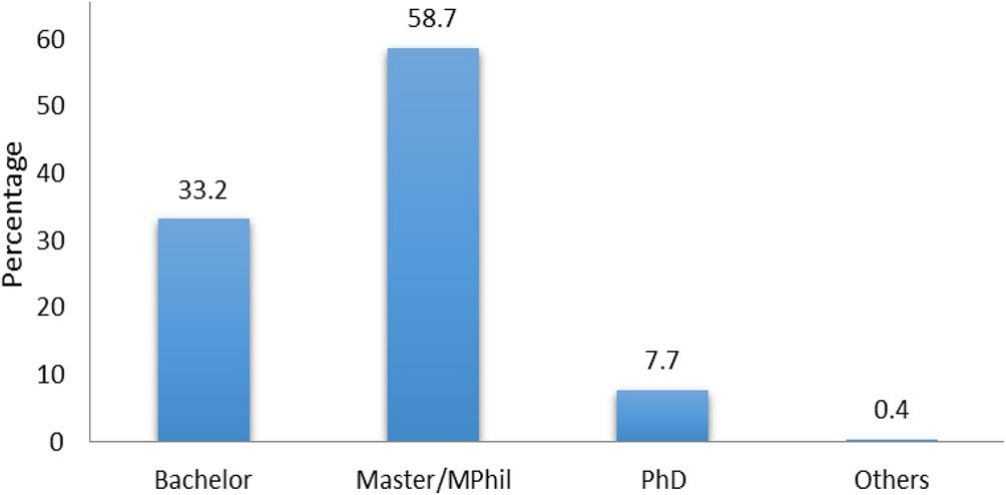
Percentage of qualification wise responses. The bar chart represents percentages of respondents having different qualifications.

**Figure 4:**
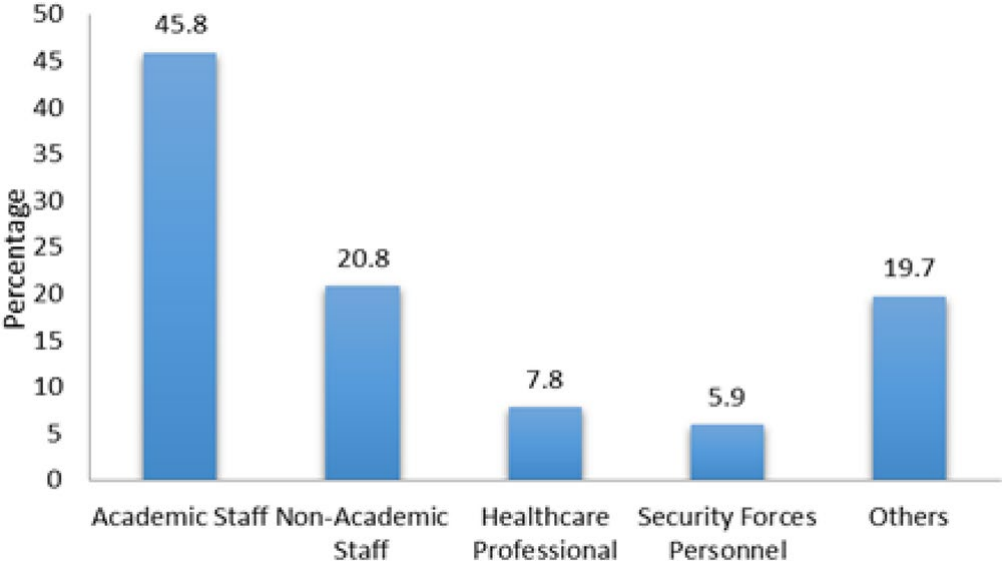
Percentage of profession wise responses. The bar chart represents percentages of respondents having different professions.

A total of 1132 respondents comprising 331 (29.2%) females and 799 (70.6%) males participated in this survey (Fig. 1). Participants were divided into four age groups, group I was < 25 years, group II was 25–40 years with the highest response percentage (62.9), group III was 41–60 years and group IV was > 60 years with the least response percentage (1.3) (Fig. 2). Survey participants were divided into four groups on the basis of educational level, bachelor (33.2%), Master/MPhil with the highest response percentage (58.7), PhD (7.7%) and only 0.4% response was obtained from others (Fig. 3). A survey response was gathered from different professions including academic staff with the highest frequency (45.8%), non academic staff (20.8%), healthcare professionals (7.8%), security forces personnel being least (5.9%) and others (19.7%) (Fig. 4).

### Question wise responses and percentages

To access the basic knowledge of COVID-19, an overwhelming majority of participants (93.29%) were aware that it is a viral disease. In query regarding the prevalence of COVID-19, 39.19% reported no case in their residing area, while 23.14% reported less than 10 cases, 10.07% reported 10–50 cases, 13.34% reported more than 50 cases and 16.61% had no idea regarding it. When respondents were asked about the possible origin of COVID-19, the majority (45.41%) was of the opinion that bat is the origin and 36.75% people were not sure. In a query about seasonality of COVID-19, 65.81% of respondents agree that it is not seasonal and 10.34% were of view that it is seasonal while 22.26% did not know. Most of the respondents (70.67%) were of the view that direct contact with infected persons, cough droplets, hand shaking, visiting hospitals andpublic gathering, all could cause COVID-19 infection.

In question assessing the knowledge about common signs and symptoms of COVID-19, (77.65%) of the respondents believed that cough, fever, headache and shortness of breath are most common. Only 19.26% thought there might be symptoms other than above mentioned. In response to a query about the available vaccine of COVID-19, the majority of the study respondents (88.43%) revealed that there is no vaccine developed yet. It was interesting to know that (89.65%) of the respondents mentioned lung problems as a major complication of COVID-19.

Most of the respondents (85.07%) believed that they could prevent from COVID-19 infection by doing collective practices including hand washing, wearing masks, self quarantine and social isolation however little showed inclination to single practice self quarantine (8.22%) and hand washing (4.77%). As laid in Table 1 majority of the respondents (71.29%) were of the view that healthcare workers are at high risk of getting infected with COVID-19. However (19.26%) also considered field workers at risk.

**Table 1:** Question wise responses and percentages.

Statements	Responses	No. of respondents (%)
What is COVID-19	Bacterial disease	68 (6.01)
	Viral disease	1056 (93.29)
	Others	3 (0.27)
	No response	5 (0.44)
Number of COVID-19 patients in your area as per your knowledge	0 Case	409 (36.13)
	10–50	114 (10.07)
	Less than 10	262 (23.14)
	More than 50	151 (13.34)
	No idea	188 (16.61)
	No response	8 (0.71)
Your opinion on the possible Origin of COVID-19?	Bat	514 (45.41)
	Bat and snake	14 (1.24)
	I think COVID-19 is a "Man Made Biological Weapon"	63 (5.57)
	Not sure	416 (36.75)
	Pet animals	31 (2.74)
	Snake	26 (2.3)
	Others	51 (4.51)
	No response	17 (1.5)
Is COVID-19 seasonal?	Don’t know	252 (22.26)
	May be	10 (0.88)
	No	745 (65.81)
	Yes	117 (10.34)
	No response	8 (0.71)
What are the Possible Ways that you may get Infected with COVID-19?	Cough droplets	9 (0.8)
	Cough droplets, hand shaking, quarantine areas, hospitals and public gathering	940 (83.04)
	Direct contact with infected person	81 (7.16)
	Hand shaking and public gathering	21 (1.86)
	Hospitals	5 (0.44)
	Others	5 (0.44)
	Public gathering	53 (4.68)
	Quarantine areas	8 (0.71)
	Through air	10 (0.88)
What are the common symptoms of COVID-19?	Cough	18 (1.59)
	Fever, cough, flue and shortness of breath	999 (88.25)
	Flue	23 (2.03)
	Shortness of breath	86 (7.6)
	Others	6 (0.53)
Is vitamin C protective against COVID-19?	Don’t know	237 (20.94)
	No	72 (6.36)
	Yes	815 (72)
	No response	8 (0.71)
Is there any vaccine available for the cure of COVID-19?	Don’t know	94 (8.3)
	No	1001 (88.43)
	Yes	30 (2.65)
	No response	7 (0.62)
What are the possible complications of COVID-19?	Don't know	3 (0.27)
	Heart problem	10 (0.88)
	Heart problem, lungs problem and renal problem	74 (6.54)
	Lungs problem	975 (86.13)
	Lungs problem and renal problem	57 (5.04)
	No response	13 (1.15)
What are the Method(s) that could be useful in preventing COVID-19?	Hand sanitizing/washing	54 (4.77)
	Self-quarantine	93 (8.22)
	Wearing masks	11 (0.97)
	Wearing masks, hand sanitizing/washing, disinfection, social distancing, self quarantine	963 (85.07)
	No response	11 (0.97)
Which occupation is at the most risk of getting COVID-19?	Academic professionals	42 (3.71)
	Armed forced	35 (3.09)
	Field workers	212 (18.73)
	Field workers, Health care workers, Academic professionals, Armed forced	14 (1.24)
	Health care workers	815 (72)
	No response	14 (1.24)
How often you disinfect/wash hands in a day?	After every hour	559 (49.38)
	After every hour, as and when I come back from outside	1 (0.09)
	After every hour, as and when I come back from outside, before eating	477 (42.14)
	Before eating	59 (5.21)
	Rarely	19 (1.68)
	No response	17 (1.5)
How often you take shower?	Daily	773 (68.29)
	Once a week	22 (1.94)
	Thrice a week	218 (19.26)
	Twice a week	97 (8.57)
	No response	22 (1.94)
You got the initial information about COVID-19 through	Facebook	55 (4.86)
	Friends	20 (1.77)
	Friends, official apps/government databases, Email, WhatsApp, Facebook, Telecommunication Authorities	356 (31.45)
	Newspaper, TV	329 (29.06)
	Official Apps/Government databases	28 (2.47)
	Telecommunication authorities	10 (0.88)
	TV, Friends, WhatsApp, Facebook	58 (5.12)
	TV, Official Apps/government databases	66 (5.83)
	TV, WhatsApp	124 (10.95)
	WhatsApp, Facebook	60 (5.3)
	Others	7 (0.62)
	No response	19 (1.68)
Spread of COVID-19 has restricted my mobility	Agree	261 (23.06)
	Disagree	14 (1.24)
	Neutral	73 (6.45)
	Strongly agree	756 (66.78)
	Strongly disagree	8 (0.71)
	No response	20 (1.77)
Wearing masks is compulsory to avoid COVID-19	Agree	326 (28.8)
	Disagree	83 (7.33)
	Neutral	142 (12.54)
	Strongly agree	557 (49.2)
	Strongly disagree	5 (0.44)
	No response	19 (1.68)
Public events; like religious gatherings, musical festivals or sport should be avoided these days	No	26 (2.3)
	Yes	1085 (95.85)
	No response	21 (1.86)
Satisfied with the government arrangements for screening people at airports, sea ports and entry points	Neutral	215 (18.99)
	Satisfied	334 (29.51)
	Unsatisfied	141 (12.46)
	Very dissatisfied	107 (9.45)
	Very satisfied	316 (27.92)
	No response	19 (1.68)
You feelings about public awareness campaigns on COVID-19 by the government	Neutral	144 (12.72)
	Satisfied	534 (47.17)
	Strongly dissatisfied	19 (1.68)
	Strongly satisfied	335 (29.59)
	Unsatisfied	72 (6.36)
	No response	28 (2.47)
Government/institutional response and arrangements to protect community from COVID-19 outbreak?	Don’t meet my expectations	173 (15.28)
	Meet my expectations	312 (27.56)
	Somewhat meet my expectations	620 (54.77)
	No response	27 (2.39)
Do closing institutions/lock-down pose any effect on your productivity?	No	279 (24.65)
	Yes	827 (73.06)
	No response	26 (2.3)
Current situation is stressful to you	Moderately stressful	581 (51.33)
	Not stressful	115 (10.16)
	Very stressful	412 (36.4)
	No response	24 (2.12)
Staying at home caused any of the following situation(s) in me	Anxiety/depression, fatigueless	274 (24.2)
	Anxiety/depression, fear, blood pressure issues, fatigueless	229 (20.23)
	Fatigueless	97 (8.57)
	Fear	115 (10.16)
	Loneliness, fatigueless	153 (13.52)
	None	99 (8.75)
	No response	165 (14.58)
I Relax myself in current situation of COVID-19 by	Do religious practices	118 (10.42)
	Do religious practices, play indoor game (individually)	55 (4.86)
	Play indoor game (individually)	33 (2.92)
	Sleep most of the time	100 (8.83)
	Sleep most of the time, watch TV/social media/news, do religious practices, play indoor game (individually)	299 (26.41)
	Watch TV/social media/news	256 (22.61)
	Watch TV/social media/news, do religious practices, play indoor game (individually)	154 (13.6)
	Others	78 (6.89)
	No response	39 (3.45)
When the COVID-19 may be over from your area, your perception?	May persist long	251 (22.17)
	No idea	448 (39.58)
	Very soon	400 (35.34)
	Will persist long	8 (0.71)
	No response	25 (2.21)

In present study two questions were asked to assess the hygiene practices among people during the COVID-19 outbreak, hourly hand washing practice was reported by (50.18%) and (41.43%) of the respondents said that they are likely to practice it when they come home. With regards to personal hygiene 68.20% reported daily bathing.

When people were asked about the source of information about COVID-19, (29.06%) of respondents stated that newspaper and TV were the most trusted source, (31.45%) rely on friends, official apps, government databases, emails, social media and telecommunication authorities (10.95%). When asked about restricted mobility during COVID-19 majority (66.78%) were strongly agreed and (23.06%) were agreed to the statement. From the data collected it is evident that half of the respondents (49.20%) strongly agree to consider masks as a compulsory tool to avoid COVID-19. To evaluate the attitude of people to public events, responses showed that (95.85%) people avoid religious gatherings, musical festivals and sport events during the COVID-19 outbreak in the country.

In query regarding satisfaction on Government arrangements for screening people at entry ports overall (21.91%) respondents showed disagreement while more than half (57.43%) reported satisfaction. When asked about public awareness campaigns on COVID-19 by the government, an overall (74.83%) response was satisfactory. Only a few (6.01%) of respondents were unsatisfied.

A prevailing myth is that Vitamin C can be directly protective against COVID-19, our study got 72% responses in affirmation, only few responses (6.36%) in No and 20.94% were unaware about it. In a query regarding government/institutional response and arrangements to protect the community from COVID-19, most of the respondents (27.56%, 54.77%) showed their trust on these activities. However, dissatisfaction was also expressed by (15.28%) by saying that government/institutional responses do not meet their expectations. A large number (73.06%) of the participants of study stated that closure of their institutions/lockdown to contain COVID-19 has affected their productivity.

In our study more than half of the respondents (51.33%) said that the current situation is moderately stressful while for (36.40%) it is stressful. Response of (10.16%) showed that they are not affected by the current situation of COVID-19. Partial lock down is implemented in the country due to the pandemic situation of COVID-19, our study assessed that staying at home caused anxiety, depression and fatigue in (24.2%) of participants while (20.23%) expressed fear and blood pressure issues along with above mentioned problems, 13.52% fear and loneliness and 10.16% expressed only fear. The overall observation of the activities revealed that while staying at home (44.52%) people spend time sleeping, on social media and TV news. While (55.48%) were engaged in other activities. A query “when the COVID-19 may be over from your area? Got mixed responses, (22.88%) said that it will persist long, (36.40%) showed no idea and (33.92%) said that it will go away very soon.

### Distribution of responses with respect to age, gender, educational level and occupation

In Table 2 we considered the responses to the questions with respect to age, gender, educational level and occupation. Pearson chi-square test showed the chi-square values and significant mean difference for each question. Mean difference value of 0.05 or less was considered statistically significant. Variation and similarity of responses were assessed by this test. With respect to basic knowledge of COVID-19, significant variation was observed with age group (0.000*) Query regarding the prevalence of COVID-19 got significant variations for all variables except gender responses (0.083), Significant variation was observed with respect to query regarding the possible origin of COVID-19 in gender and occupation responses (0.009*, 0.013*). A query about seasonality of COVID-19 got significant results only in educational group responses (0.000*).

**Table 2:** Pearson Chi-square tests.

Statements		Age	Gender	Educational level	Occupation
What is COVID-19	Chi-square	52.622	7.200	18.034	17.151
	Sig	0.000*	0.303	0.261	0.144
Number of COVID-19 patients in your area as per your knowledge	Chi-square	32.757	16.640	292.033	33.259
	Sig	0.005*	0.083	0.000*	0.032*
Your opinion on the possible origin of COVID-19?	Chi-square	28.926	29.438	44.689	47.362
	Sig	0.116	0.009*	0.126	0.013*
Is COVID-19 seasonal?	Chi-square	23.63	4.905	135.891	21.775
	Sig	0.072	0.897	0.000*	0.353
What are the possible ways that you may get infected with COVID-19?	Chi-square	49.628	33.385	99.538	46.9
	Sig	0.002*	0.007*	0.000*	0.043*
What are the common symptoms of COVID-19?	Chi-square	41.934	37.441	110.186	30.978
	Sig	0.004*	0.001*	0.000*	0.318
Is vitamin C protective against COVID-19?	Chi-square	14.193	8.349	78.59	33.663
	Sig	0.116	0.214	0.000*	0.001*
Is there any vaccine available for the cure of COVID-19?	Chi-square	30.06	9.884	42.53	17.339
	Sig	0.000*	0.13	0.000*	0.137
What are the possible complications of COVID-19?	Chi-square	20.319	0.989	49.692	19.13
	Sig	0.16	1	0.002*	0.513
What are the method(s) that could be useful in preventing COVID-19?	Chi-square	28.961	15.97	46.232	30.064
	Sig	0.004*	0.043*	0.001*	0.018*
Which occupation is at the most risk of getting COVID-19?	Chi-square	21.371	7.985	59.146	37.776
	Sig	0.125	0.63	0.000*	0.009*
How often you disinfect/wash hands in a day?	Chi-square	28.119	39.145	31.686	22.502
	Sig	0.021*	0.000*	0.167	0.314
How often you take Shower?	Chi-square	24.995	87.276	29.669	29.564
	Sig	0.015*	0.000*	0.075	0.020a*
You got the initial information about COVID-19 through	Chi-square	36.805	27.807	119.634	59.561
	Sig	0.297	0.182	0.000*	0.059
Spread of COVID-19 has restricted my mobility	Chi-square	75.168	7.141	54.415	41.766
	Sig	0.000*	0.712	0.001*	0.003*
Wearing masks is compulsory to avoid COVID-19	Chi-square	35.004	6.691	34.043	24.749
	Sig	0.002*	0.754	0.107	0.211
Public events; like religious gatherings musical festivals or sport should be avoided these days	Chi-square	10.156	4.014	48.881	7.135
	Sig	0.118	0.404	0.000*	0.522^a^
Satisfied with the government arrangements for screening people at airports sea ports and entry points	Chi-square	55.749	21.707	64.885	23.659
	Sig	0.000*	0.017*	0.000*	0.258
You feelings about public awareness campaigns on COVID-19 by the Government	Chi-square	49.381	28.465	170.648	74.416
	Sig	0.005*	0.055	0.000*	0.000*
Government/institutional response and arrangements to protect community from COVID-19 outbreak?	Chi-square	28	7.651	34.03	14.634
	Sig	0.001*	0.265	0.003*	0.262
Does closing institutions/lock-down pose any effect on your productivity?	Chi-square	15.176	3.41	24.776	6.592
	Sig	0.086	0.756	0.053	0.883
Current situation is stressful to you	Chi-square	21.343	32.453	44.974	13.617
	Sig	0.011*	0.000*	0.000*	0.326^a^
Staying at home caused any of the following situation(s) in me	Chi-square	35.795	19.42	81.849	41.436
	Sig	0.023*	0.15	0.000*	0.049*
I Relax myself in current situation of COVID-19 by	Chi-square	38.6	64.187	64.987	84.135
	Sig	0.621	0.000*	0.647	0.009*
When the COVID-19 may be over from your area your perception?	Chi-square	65.676	17.279	48.657	72.316
	Sig	0.011*	0.943	0.976	0.07

*The Chi-square is significant (two variables are not independent) at the 0.05 level.

On questions concerning possible ways of getting infected with COVID-19 there was also significant variability in responses of all variables i.e., age, gender, educational level and occupation (0.002* 0.007* 0.000* 0.043*). On the question about common signs and symptoms of COVID-19, we found significant variations in responses with respect to age, gender and educational groups (0.004* 0.001* 0.000*). Interestingly myth about prevention of vitamin C against COVID-19, gathered significant variations in responses from educational and occupational groups (0.000* 0.001*). For knowing about the vaccine availability and possible complications of COVID-19, significant variations were observed with respect to age and educational group (0.000*, 0.000*, 0.002*). It is clear from the significant responses that people are well aware of the protective measures against COVID-19 with respect to all variables in Table 2 (0.004* 0.043* 0.001 0.018*).

When asked about the occupation at risk to COVID-19, significant variations were observed from educational and occupation groups (0.000* 0.009*). In our study significant variations were observed in responses to queries about disinfecting practices with regard to age, gender and educational groups. Variation in responses were observed from educational groups (0.000*) when people were asked about the source of getting information about COVID-19. Significant variations in the responses from all variables in Table 2 showed that COVID-19 has restricted their mobility except in gender group (0.000* 0.712 0.001* 0.003*). Response from age group with significant value (0.002*) showed that for this group wearing a mask is compulsory as a protective measure against COVID-19. We observed significant variation in responses (0.000*) from different educational groups when they were asked to avoid public gatherings to contain COVID-19.

Significant variations were gathered in responses with respect to almost all variables when participants were asked about the government arrangements taken for screening at entry point, people awareness campaigns and government and institutional response to protect the community from COVID-19 (Table 2). Insignificant variations in responses (0.086 0.756 0.053 0.883) showed that closing of institutes did not pose any effect on respondent’s productivity. However current situation is stressful for all variables except occupational group as shown by significant value in Table 2 (0.011* 0.000* 0.000*).

Significant and insignificant both variations were observed in the responses when participants were asked about their activities during partial lock down (Table 2). On the question when COVID-19 outbreak will go away from their area, significant variation in responses was observed with respect to age group (0.011*).

## Discussion

**Figure 5:**
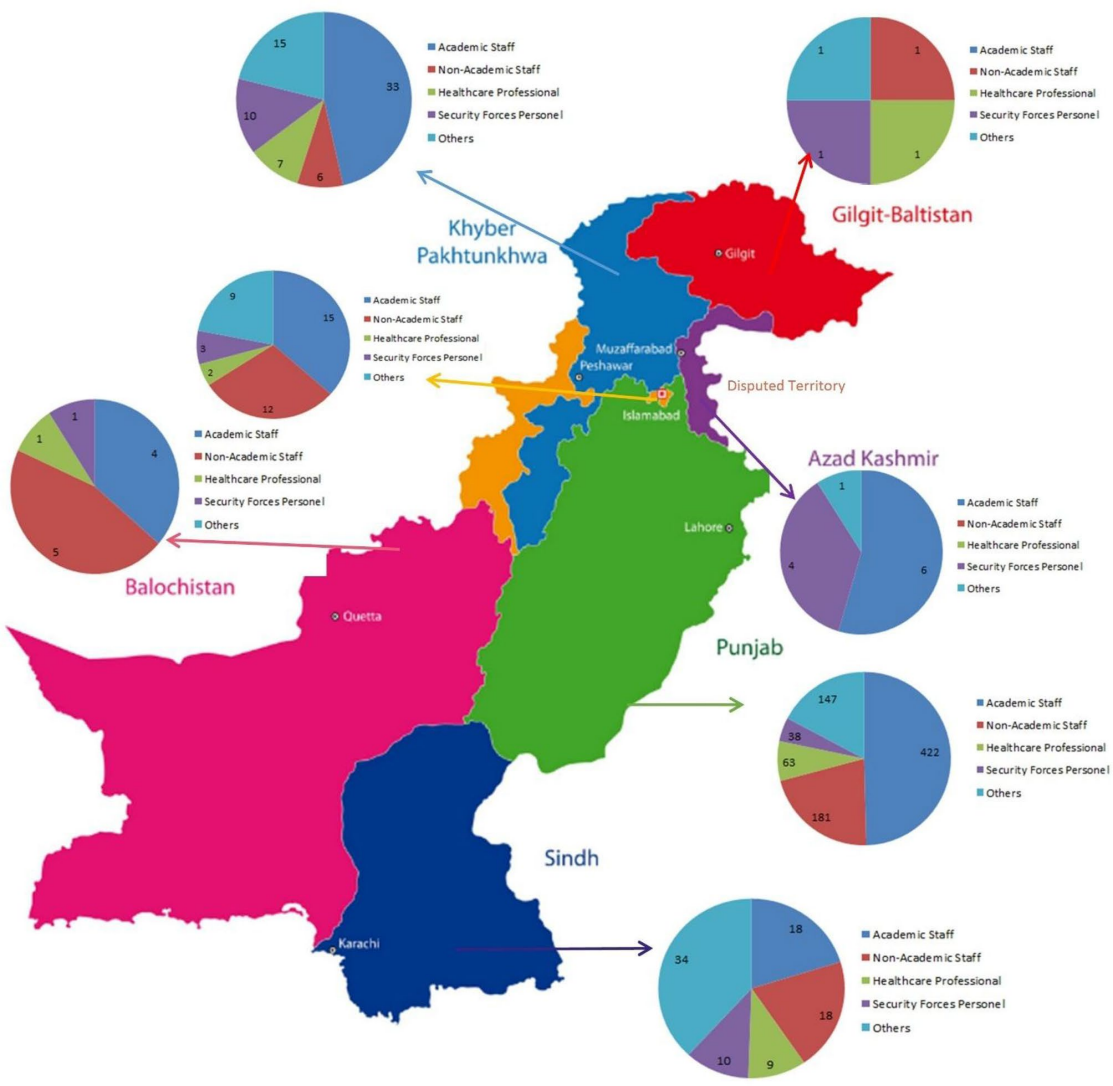
Respondents’ distribution province wise. (http://clipart-library.com/clipart/pc5oXeaoi.htm).

This study was started during the first wave (March 2020) of COVID-19 in Pakistan and authorities imposed the complete lockdown and aggressive measures to proclaim the spread of disease. Through a self-designed questionnaire, data regarding the awareness pertaining to the origin, symptoms, spread, control, and prevention of COVID-19 was collected in this study. It is important to consider that the highest number of responses were received by individuals falling in the age group of 25–40 years, having Master/MPhil as their highest education and belonging to the academic profession from different regions of Pakistan (Fig. 5). Results indicate a high level of right awareness among individuals, especially regarding the cause (93.29%), the routes of transmission (70.76%), the signs and symptoms (77.65%), preventive strategies (~ 85%) and health complications due to the infection (89.65%). A similar study has conducted in Punjab revealed the satisfactory response of health care professionals regarding COVID-19 awareness, spread, signs and symptoms and complications. This may be attributed to the authorities who persistently provided COVID-19 information to not only health professionals^12^ but to general public too.

Interestingly, the source of information for the respondents regarding COVID-19 are predominantly modern media (60.51%). This is supported by another study conducted in Pakistan which reported that majority of the respondents of study used social media as their major source of information^13^. A fear of COVID-19 is a major mental health problem not only in Pakistan but around the globe. It is key to understand that the outbreak itself, and the consequential lockdown, has caused significant psychological impact on the society with 87.73% people reporting the situation to be stressful, leading them to face anxiety, depression and fatigue. This fear may be attributed to closure of educational institutes, private hospitals and ban on intra and intercity transport^14^.

According to our results, the percentage incidence of new cases reported per day have reduced since the first few cases and the launch of the National Action Plan in Pakistan. However, this factor is independent of the fact that there are limited screening facilities available in the country. On one hand, sharing of border and vast trade relations with China where COVID-19 initiated and Iran which has a high number of infected individuals, and lack of ample medical facilities and health awareness in Pakistan on the other hand, the country is in a unique situation^15^. In view of the lack of appropriate prevention and control policies at educational and medical organizations, effective awareness campaigns at both national and community level were launched under the National Action Plan for COVID-19 proposed by the Ministry of Health Services, Regulation and Coordination, Government of Pakistan^10^. This study conducted almost a month after the first reported COVID-19 case in Pakistan aimed at evaluating the general awareness of professionals regarding this pandemic. Results reported also have an outcome of assessing effectiveness of the National Action Plan and could be used as a pointer for further course of action.

## Methods

### Data collection

The data presented in this paper was obtained one month after the outbreak of COVID-19 in Pakistan as the first case was reported on February 26, 2020. Information about COVID-19 outbreak was obtained by a self-designed questionnaire that was distributed via online portal of Virtual University of Pakistan, emails and other sources of communication (mainly WhatsApp groups) among professionals of different institutions across the country. The study was approved by the Departmental Ethical Committee of the Department of Molecular Biology, Virtual University of Pakistan, which conforms tothe Declaration of Helsinki and all methods in this study were carried in accordance with relevant guidelines. Questionnaire was developed to assess the awareness about the basic knowledge, prevalence in the residing area, mode of transmission of infection, common signs and symptoms, complications, preventive measures, effects on personal health, perceptions, government response and arrangement on COVID-19 outbreak. The questionnaire was filled by individuals voluntarily with a prior informed consent taken in the form of a covering letter shared along with the questionnaire.

In the current scenario respondents from different cities of Pakistan were included in this study. Demographic data including age, gender, educational level and occupation of the participants were recorded. Query was addressed about the number of infected people in the residing area of the participants. In the questionnaire, respondents were inquired about the origin, common signs and symptoms and the possible ways to get infected with COVID-19. At the time of this global emergency several myths have been circulated about COVID-19, participants were asked about the cure, vaccination and seasonality of its spread. Questions investigating the respondents’ knowledge about infection complications, effective preventive measures and common practices that need to be done in this pandemic were assessed. Questionnaire also contained questions about personal hygiene to analyze personal health efforts.

Correlation of people’s behavior towards the current partial lockdown situation in the country was evaluated by different questions. To evaluate the risk of social and religious gatherings in the present scenario, respondents were asked whether they avoid these public events or not? A range of questions was included in the questionnaire on their opinion of the awareness campaigns, arrangement of screening, preventive strategies and policies at national and institutional level.

To those who are restricted to move out due to quarantine to avoid viral spread, a series of questions was asked to evaluate the level of stress and activities they are doing in this time of public health crises. A partial lockdown strategy has been applied to almost all institutes across the country, a question was also asked regarding productivity of the respondents. Within the questionnaire respondents were asked which source of information they rely for the latest update of COVID-19 across the country as well as globe.

To avoid the community transfer social distancing strategy has been implemented in almost all institutions and in this situation online mode of education has been launched to reduce the knowledge gap. People’s response was recorded on effectiveness of online mode of education in their working institutes.

For this study only one response was accepted from one participant and incomplete responses were excluded. Current faculty, academic, non-academic staff, healthcare professionals and security professionals were included in this survey.

### Statistical analysis

Data obtained was statistically analyzed by computer software SPSS version 21. Results were presented in frequency and percentage. Pearson chi-square test was performed to analyze the mean difference between variables.

## Conclusion

Deeming public awareness to be crucial in preventing the spread of COVID-19, which otherwise lacks effective treatment and preventive measures, vast public awareness campaigns played a key role in the fight against it.The awareness, attitude and practices for the survival of humans have been influenced by the COVID-19 pandemic. We being the residents of the developing country are still fighting against COVID-19 and for the ultimate accomplishment, community should follow the public health measures. The current study reveals the level of awareness regarding knowledge, attitude and preventative practices against COVID-19 among professionals from different fields in Pakistan.The results of our study reveal that public awareness campaigns under the National Action Plan for COVID-19 of the Pakistani government have led to significant awareness of the young, educated and professional class in Pakistan. It was evident that the community’s overall response was satisfactory with respect to awareness about the symptoms, mode of transmission and precautionary measures of COVID-19. And this positive approach of public also influences the practices towards COVID-19 as the majority of the people showed their willingness to follow the guidelines set by the government.
